# Antioxidant Capacity of the Leaf Extract Obtained from *Arrabidaea chica* Cultivated in Southern Brazil

**DOI:** 10.1371/journal.pone.0072733

**Published:** 2013-08-29

**Authors:** Jackeline Tiemy Guinoza Siraichi, Daniele Fernanda Felipe, Lara Zampar Serra Brambilla, Melissa Junqueira Gatto, Vânia Aparecida Terra, Alessandra Lourenco Cecchini, Lucia Elaine Ranieri Cortez, Edson Rodrigues-Filho, Diógenes Aparício Garcia Cortez

**Affiliations:** 1 Pharmaceutical Sciences Postgraduate Program, Universidade Estadual de Maringá, Maringá, State of Paraná, Brazil; 2 Department of Pharmacy, Universidade Estadual de Maringá, Maringá, State of Paraná, Brazil; 3 Instituto Federal do Paraná, Londrina, State of Paraná, Brazil; 4 Department of Pathological Sciences, Universidade Estadual de Londrina, Londrina, State of Paraná, Brazil; 5 Postgraduate Program in Health Promotion, Centro Universitário de Maringá, State of Maringá, Paraná, Brazil; 6 Department of Chemistry, Universidade Federal de São Carlos, São Carlos, State of São Paulo, Brazil; University of New South Wales, Australia

## Abstract

*Arrabidaea chica* leaf extract has been used by people as an anti-inflammatory and astringent agent as well as a remedy for intestinal colic, diarrhea, leucorrhea, anemia, and leukemia. *A. chica* is known to be a good producer of phenolics. Therefore, in the present study, we investigated its antioxidant activity. The phenolic composition of *A. chica* leaves was studied by liquid chromatography coupled to diode array detection (LC–DAD) and liquid chromatography coupled to electrospray ionization-tandem mass spectrometry (LC–ESI-MS/MS), and isoscutellarein, 6-hydroxyluteolin, hispidulin, scutellarein, luteolin, and apigenin were identified. The extract from leaves of *A. chica* was tested for antioxidant activity using the 2,2-diphenyl-1-picrylhydrazyl (DPPH) method, β-carotene bleaching test, and total reactive antioxidant potential (TRAP) method. The crude extract quenched DPPH free radicals in a dose-dependent manner, and the IC_50_ of the extract was 13.51 µg/mL. The β-carotene bleaching test showed that the addition of the *A. chica* extract in different concentrations (200 and 500 µg/mL) prevented the bleaching of β-carotene at different degrees (51.2% ±3.38% and 94% ±4.61%, respectively). The TRAP test showed dose-dependent correlation between the increasing concentrations of *A. chica* extract (0.1, 0.5, and 1.0 µg/mL) and the TRAP values obtained by trolox (hydro-soluble vitamin E) 0.4738±0.0466, 1.981±0.1603, and 6.877±1.445 µM, respectively. The 2 main flavonoids, scutellarein and apigenin, were separated, and their antioxidant activity was found to be the same as that of the plant extract. These 2 flavonoids were quantified in the plant extract by using a validated HPLC-UV method. The results of these tests showed that the extract of *A. chica* had a significant antioxidant activity, which could be attributed to the presence of the mixture of flavonoids in the plant extract, with the main contribution of scutellarein and apigenin.

## Introduction

Flavonoids are a class of secondary plant metabolites having diverse structures (about 9,000) [1]. They are polyphenolic compounds derived from 2-phenylchromane, which is commonly found in many plants, vegetables, and flowers [2]. Numerous studies have focused on determining the biological and pharmacological activities of flavonoids that are thought to have beneficial effects on human health. The flavonoids exhibit several beneficial activities, including antibacterial, antiviral, anti-inflammatory, antioxidant, anticarcinogenic, antiallergenic, hepatoprotective, vasodilating, and antithrombotic activities. Most research has been focused on their antioxidant potential, which is due to their ability to reduce free radical formation and scavenge free radicals, leading to a therapeutic possibility of being used against free radical-mediated diseases [3]–[5].

There is a growing interest in natural antioxidants present in medicinal and dietary plants that might help attenuate oxidative damage [6]. Furthermore, there is an increasing economic interest in natural resources such as herbal extracts that contain antioxidants for use in cosmetic science as beauty products and to maintain the physiological balance of the human skin [7]. To identify antioxidants in herbs, we investigated the antioxidant potential of *Arrabidaea chica*, a shrub plant that shows widespread distribution from South Mexico to central Brazil, particularly in the Amazon region. This plant belongs to the family Bignoniaceae which contains approximately 120 genera and 800 species [8]–[10]. It has been used as an anti-inflammatory and astringent agent as well as a remedy for intestinal colic, diarrhea, leucorrhea, anemia, and leukemia. A literature review indicated that this plant is a source of anthocyanins, flavonoids, tannins, and phytosterols [11]–[14].

Therefore, this study aimed to analyze 2 major flavonoids obtained from the leaf extract of *A. chica* by using high-performance liquid chromatography (HPLC) and investigate the antioxidant potential of the extract by using the 2,2-diphenyl-1-picrylhydrazyl (DPPH) method, β-carotene bleaching test, and total reactive antioxidant potential (TRAP) test, and to compare the activity of the extract with that of the 2 metabolites separately using the TRAP method.

## Material and Methods

### 2.1. Reagents and Chemicals

For HPLC analysis, methanol (HPLC grade; Mallinckrodt Baker, S.A., México) and ultrapure water (Milli-Q system; Millipore, Bedford, USA) were used as the mobile phase. Apigenin and scutellarein (Merck, Darmstadt, Germany) were used as the external standards. The antioxidant systems, β-carotene, 2,2-diphenyl-1-picrylhydrazyl (DPPH), butylated hydroxytoluene (BHT), and quercetin were purchased from Sigma-Aldrich (Saint Louis, MO, USA). Chloroform, 2,2-azobis (2-amidinopropane) dihydrochloride (ABAP), Trolox, and Tween 80 were purchased from Merck (Darmstadt, Germany).

### 2.2. Plant Material

The matrix plant of *A. chica* was obtained from Colorado d’Oeste (RO) in February 2008, and the seedlings were cultivated at the “Prof. Irenice Silva” medicinal plant garden of the Maringá State University, PR, Brazil, by using only organic fertilizers. The samples of *A. chica* leaves were collected after 2 years of cultivation. A voucher specimen (number HUM 11428) was deposited at the Herbarium of the Maringá State University. *A. chica* is one of the medicinal plants of interest to the National Health Care System in Brazil and has been registered under IBAMA-SISBIO as 1945130.

### 2.3 General Experimental Procedures

The nuclear magnetic resonance (NMR) spectra were obtained using VARIAN GEMINI300 (7.05 T) spectrophotometer by using tetramethylsilane (TMS) as the internal standard. Mass spectrometer: low-resolution data were collected using a triple quadrupole Micromass Quattro LC instrument equipped with a “Z-spray” ion source. Column chromatography (CC) was performed using silica gel 60 (70–230 and 230–400 mesh) and Sephadex-20. Thin layer chromatography (TLC) was performed using silica gel plates F_254_ (0.25 mm in thickness).

### 2.4. Extraction and Isolation

Leaves were collected from 30 plants of *A. chica,* dried, blended, and powdered using a knife-mill (350 g). The extract was obtained using the maceration method with ethanol-water (9∶1 v/v). The extract was filtered, and the organic solvent was removed under vacuum at 40°C and lyophilized, resulting in a red residue (5.72 g). The residue (1 g) was dissolved in 10 mL of ethanol/water (6∶4; v/v) and chromatographed using Sephadex LH-20 eluting with ethanol-water (9∶1; v/v) to yield 25 fractions of 10 mL each. In vitro bioguided fractionation of the extract for antioxidant content was conducted using total reactive antioxidant potential (TRAP) method.

The most active fractions were analyzed using UV spectroscopy and TLC; the plates were developed using chloroform-methanol (75∶5; v/v) and subsequently sprayed with 2% vanillin sulfuric acid for detection. Fractions 7 and 8 yielded scutellarein (**1**) (15 mg) and fraction 9 afforded apigenin (**2**) (10 mg). The compounds were identified by ESI-MS-MS, ^1^H- and ^13^C-NMR spectral data, and by comparison with data available in the literature [11], [12], [15]–[20].

(**1**) Scutellarein

^1^H NMR (300 MHz-Acetone-d_6_) δ: 12.89 (*brs*, 5-OH, 1H); 7.93 (*d*, *J = *9.0 Hz, H-2′ and H-6′, 2H); 7.02 (*d*, *J = *9.0 Hz, H-3′ and H-5′, 2H); 6.61 (*s*, H-3, 1H); 6.48 (*s*, H-8, 1H). ^13^C NMR (75 MHz-Acetone-d_6_) δ: 183.5 (C-4); 163.4 (C-2); 161.9 (C-4′); 153.6 (C-7); 148.3 (C-9); 146.2 (C-5); 129.3 (C-6); 129.2 (C-2′ and C-6′); 121.8 (C-1′); 116.9 (C-3′ and C-5′); 105.5 (C-10); 103.5 (C-3); 94.7 (C-8). ESI-MS-MS *m/z* (rel. int.): see Table 1.

**Table 1:** LC–DAD–ESI-MS/MS identification of the major constituents of *Arrabidaea chica* (AC) extract.

N	tR	DAD	M-H^−^	Fragments (MS/MS)	Structural assignment
3	5.88	282; 340	285.3	285.3 (100); 239.3 (50); 213.2 (30); 167.1 (55); 165.1 (25); 167.1 (60);139.3 (30); 137.1 (80); 123.1 (50); 119.1 (52); 117.1 (50); 93.2 (27)	Isoscutellarein
4	6.38	277; 335	493.7	493.6 (55); 317.5 (19); 183.1 (17); 175,4 (47); 113.1 (100); 85.2 (17)	Unidentified
5	7.83	282; 346	301.3	301.3 (55); 165.1 (35); 167 (19); 139.1 (17); 137.1 (40); 133.1 (100);109.1 (17)	6-Hydroxyluteolin
6	9.07	274; 331	299.6	299.9 (8.0); 284.2 (100); 255.5 (14); 200.1 (15); 166.2 (29); 117 (10);110.2 (20)	Hispidulin (6-methylscutellarein)
1	9.80	284; 339	285.5	285.3 (46); 239.1 (14); 167.1 (25); 165.1 (35); 139.1 (25); 137.1 (54);123.0 (10); 119.2 (48); 117.2 (100); 93.1 (20)	Scutellarein
7	11.7	256; 350	285.5	285.3 (50); 199.2 (15); 175.1 (18); 151.1 (34); 149.0 (15); 133.1 (100);107.1 (17)	Luteolin
2	13.7	268; 339	269.5	269.2 (10); 151.1 (20); 117.1 (100); 107.1 (25); 65.1 (21)	Apigenin

(**2**) Apigenin

^1^H NMR (300 MHz-Acetone-d_6_) δ: 13.01 (*brs*, 5-OH, 1H); 7.94 (*d*, *J = *9.0 Hz, H-2′ and H-6′; 2H); 7.02 (*d*, *J = *9.0 Hz, H-3′ and H-5′; 2H); 6.58 (*s*, H-3, 1H); 6.54 (*d*, *J = *2.1 Hz, H-8; 1H); 6.22 (*d*, *J = *2.1 Hz, H-6; 1H). ^13^C NMR (75 MHz-Acetone-d_6_) δ: 182.3 (C-4); 164.1 (C-2); 163.9 (C-7); 161.5 (C-5); 161.2 (C-4′); 157.2 (C-9); 128.4 (C-2′ and C-6′); 121.3 (C-1′); 115.9 (C-3′ and C-5′); 105.5 (C-10); 102.5 (C-3); 99.8 (C-6); 95.0 (C-8). ESI-MS-MS *m/z* (rel. int.): see Table 1.

### 2.5. HPLC Analysis

#### 2.5.1. Sample preparation

To prepare the stock solutions, apigenin and scutellarein as well as the extract obtained from leaves of *A. chica* were dissolved in methanol, at a concentration of 1,000 µg/mL, 1,000 µg/mL, and 10,000 µg/mL, respectively. The solutions were filtered through a 0.45-mm membrane filter (Millipore, São Paulo, Brazil) prior to further analysis.

#### 2.5.2. LC–UV quantitative analysis

The analyses were carried out using a Waters Binary HPLC Pump 1525, equipped with UV-VIS detector 2489, an autosampler 2707 with a 20-µL loop, and controlled by Breeze 2 Software. A YMC-Pack Pro C18 column (i.d., 5 µm; 150 × 4.6 mm) maintained at room temperature (25°C) was used in the chromatographic analysis.

The separation was carried out in a gradient system by using a mixture of methanol:water containing 0.3% formic acid as the mobile phase. The gradient system was as follows: 50∶50 v/v to 70∶30 (0–10 min), 70∶30 v/v (10–15 min), 70∶30 to 100% methanol (15–16 min), and 50∶50 v/v (17–20 min). The detection was made at 335 nm, and the running time and flow rate were 20 min and 1.0 mL/min, respectively. The sample injection volume was 20 µL. Three determinations were accomplished for each sample. The statistical analyses of the data were performed using GraphPad Prisma Software (version 5.0, U.S.A.). The data used for statistical evaluation were relative to the quantification of apigenin and scutellarein calculated on the basis of the values of the peaks areas. Values less than 0.05 were considered statistically significant.

The liquid chromatography (LC)-UV analyses were performed using a Waters liquid chromatography system, equipped with a quaternary solvent delivery system, a Waters 600 controller, 2 Waters 600 pumps, a manual injection valve (Rheodyne) with a 20-µL loop, and a Waters 2998 PDA detector; the data were recorded using Empower software (Waters).

#### 2.5.3. LC–MS/MS and LC–DAD qualitative analysis

The Micromass Quattro LC mass spectrometer equipped with 2 quadrupole mass analyzers and an ESI ion source were used to perform ESI-MS and ESI-MS/MS analyses, which were controlled using Masslink 4.1 software. HPLC was performed using a Waters Separations Module 2695 equipped with Photodiode Array detector 2996. A YMC-Pack Pro C18 column (i.d., 5 µm; 150 × 4.6 mm) maintained at room temperature (25°C) was used for the chromatographic analysis. The separation was carried out by using water and methanol containing 0.001% formic acid as the mobile phase. Acetonitrile (100%) was used to clean the column between analyses. The gradient system was as follows: 60∶40 v/v to 40∶60 (0–10 min), 40∶60 v/v (10–15 min), 40∶60 v/v to 30∶70 v/v (15–17 min), and 100% acetonitrile (17–25 min). The absorbance was read at 335 nm, and the running time and flow rate were 25 min and 1.0 mL/min, respectively. The sample injection volume was 40 µL.

### 2.6. Validation Parameters

#### 2.6.1. Linearity

The linearity of the calibration curve for apigenin and scutellarein was established by the external standard method. Stock standard solution of apigenin at a concentration of 1,000 µg/mL was dissolved with methanol yielding concentrations of 15.63 µg/mL, 31.25 µg/mL, 62.5 µg/mL, 125 µg/mL, 250 µg/mL, and 500 µg/mL. Three determinations were carried out for each concentration. The calibration curves were obtained by plotting the peak area of apigenin versus the concentration of the standard solutions. The statistical parameters of the calibration curve–slope, intercept, and correlation coefficient–were calculated by linear regression analysis. The same procedure was followed for scutellarein; the concentrations with methanol were 31.25 µg/mL, 62.5 µg/mL, 125 µg/mL, 250 µg/mL, and 500 µg/mL.

#### 2.6.2. Precision

The repeatability (intra-day) and intermediate precision (inter-day, on 2 non-consecutive days) of the method were evaluated. The standard solutions were analyzed at 3 concentrations: 15.63 µg/mL, 62.5 µg/mL, and 500 µg/mL for apigenin, and 31.25 µg/mL, 125 µg/mL, and 500 µg/mL for scutellarein. Three determinations were carried out for each solution. The relative standard deviation (RSD, %) within the measurements of the concentrations of apigenin and scutellarein was used to evaluate the repeatability and intermediate precision.

#### 2.6.3. Limit of quantification and detection

The limit of quantification (LOQ) and limit of detection (LOD) were determined from the calibration curve of the standard apigenin and scutellarein. The LOQ and LOD were measured on the basis of a signal-to-noise ratio at about 10 and 3, respectively.

#### 2.6.4. Recovery

A recovery test of the leaf extract (10,000 µg/mL) with known content was performed by adding standard solutions at the 3 concentration levels: 15.63 µg/mL, 62.5 µg/mL, and 250 µg/mL for apigenin and 31.25 µg/mL, 125 µg/mL, and 500 µg/mL for scutellarein. The recovery was calculated as a percentage by subtracting the values obtained for the control matrix preparation from those of samples that were prepared with the added standards, divided by the amount added, and then multiplied by 100. Three determinations were carried out for each concentration.

### 2.7. Antioxidant Capacity

#### 2.7.1. DPPH method

Antioxidant activity index (AAI) was evaluated by measuring the DPPH radical scavenging potential of the extract [21]. The extract was diluted in 3 mL of methanol, yielding concentrations of 1 µg/mL, 5 µg/mL, 10 µg/mL, 15 µg/mL, 20 µg/mL, and 25 µg/mL. Next, 300 µL DPPH solution (mM) was added to these solutions. The methanolic solution of BHT (1–25 µg/mL) added to 500 µL of DPPH solution was used as the positive control. The methanolic solution containing 3 mL of methanol and 300 µL of DPPH solution was used as the negative control. The mixture was stirred for 15 s and placed at room temperature in the dark for 30 min. The absorbance of the resulting solutions was measured at 517 nm by using a spectrophotometer (Thermo Scientific, Evolution 60); the measurements were carried out in triplicate.

The radical scavenging activity was described by Scherer and Godoy [22] as follows (1):
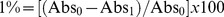
(1)where Abs_0_ was the absorbance of the blank, and Abs_1_ was the absorbance in the presence of the test compound at different concentrations.

The IC_50_ (concentration providing 50% inhibition) was calculated graphically by using a calibration curve in the linear range by plotting the extract concentration versus the corresponding scavenging effect. The antioxidant activity was expressed as the AAI, which was calculated as follows as (2): 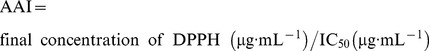
(2)


The AAI was calculated according to Scherer and Godoy [22] considering the mass of DPPH and the mass of the tested compound in the reaction, resulting in a constant for each compound, independent of the concentrations of DPPH and sample used. In this study, the plant extract was considered to show poor, moderate, strong, and very strong antioxidant activity when AAI was <0.5, 0.5–1.0, 1.0–2.0, and >2.0, respectively.

#### 2.7.2. β-carotene bleaching test

In the β-carotene/linoleic acid system, the antioxidant activity was determined on the basis of the oxidation of β-carotene induced by the oxidative degradation of linoleic acid. This test was described by Kumaran and Karunakaran [23]. For the preparation of the emulsion, β-carotene was dissolved in chloroform (0.2 mg/mL). An aliquot (2 mL) of this solution was transferred to a 100 mL flask. The chloroform was evaporated at room temperature, and then, 20 µL each of linoleic acid and Tween 80 were added. To this solution, 100 mL of hydrogen peroxide (distilled water treated with O_2_) was added, with vigorous stirring. An emulsion of β-carotene/linoleate was added to 0.2 mL of the extract at 200 and 500 µg/mL. The emulsion was placed in a water bath at 50°C for 2 h; subsequently, it was cooled down, and the absorbance was read at 470 nm. Quercetin was used as a standard. The percentage inhibition of oxidation was calculated using the following equation:
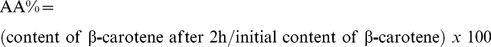
(3)


#### 2.7.3. Measurement of the total antioxidant capacity of *A. chica* extract by using TRAP method

The TRAP was measured as described by Repetto *et al.* [24] in vivo and reproduced in vitro by Pellegrini *et al*. [25], [26] and Ghiselli *et al.* [27]. This technique evaluates the levels of total antioxidants, especially of low-molecular-weight antioxidants. In this method, ABAP was used as a generator system for peroxyl radicals by thermal decomposition, producing photons that were amplified and measured by a TD 20/20 luminometer (Turner Designs, USA) via a chemiluminescence reaction. The reaction was inhibited by analogs of vitamin E (Trolox).

The antioxidant activity of the fractions obtained from *A. chica* were analyzed, and fractions 7–8 (scutellarein) and fraction 9 (apigenin) were found to be more active. Thus, the purified compounds, scutellarein and apigenin, obtained from *A. chica,* and the extract of *A. chica* at a concentration of 0.1 µg/mL, 0.5 µg/mL, and 1.0 µg/mL were mixed with 200 mM luminol and 200 mM ABAP to a final volume of 1 mL. TRAP of these samples was calculated by using the following equation:
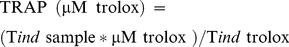
(4)where T*ind* represents the induction time for which the antioxidant produces peroxyl formation/number of photons readings in 1 min.

The TD20/20 luminometer detects chemiluminescence at a wavelength range of 300–650 nm, has a sensitivity of 50.1%, and can produce 450–12,500 readings. The luminometer was connected to a microcomputer through Spreadsheet Interface v. 1.0 program to record the chemiluminescence emission. Origin v. 8.0 software was used to plot chemiluminescence curves.

### 2.8. Statistical Analysis

The statistical analyses were performed using GraphPad Prism 5 software. The IC_50_ values were calculated using the linear regression analysis. Homogeneous data were analyzed using a one-way analysis of variance (ANOVA). The level of significance was set at 5%, and the differences were considered significant when *p*<0.05.

## Results and Discussion

### 3.1. Phytochemical Analysis

The extract obtained from *A. chica* was fractionated on Sephadex LH-20, and the corresponding fractions were assayed for antioxidant activity. NMR analysis of these fractions and 2 further purified compounds revealed that scutellarein and apigenin are the major compounds in the antioxidant active extract. To our knowledge, this is the first report of scutellarein extraction from this species. On the other hand, HNMR analyses of all fractions revealed no signal for H-3 and H-4 (9.13; *d*, *J = *8.8 Hz and 8.29; *d*, *J = *8.8 Hz, respectively) for the pigment called carajurin [12], which is typically found in this plant species.

**Figure 1:**
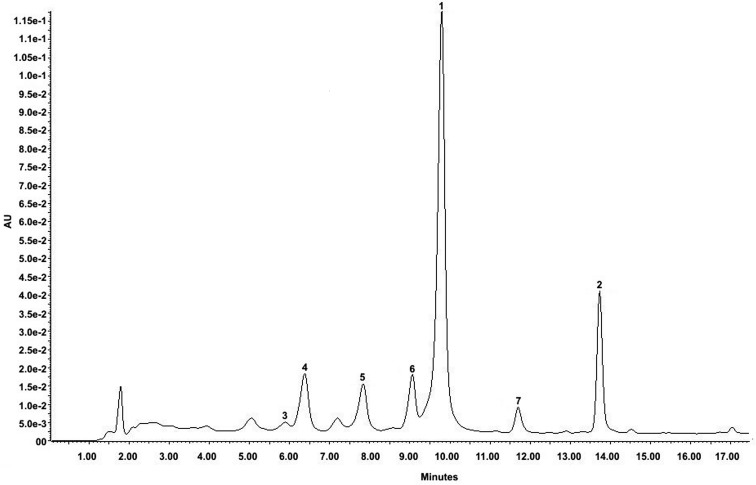
LC–DAD chromatogram at 335 nm of the hydroethanolic extract of *Arrabidaea chica* leaves. The peaks are labeled according to the compounds listed in Table 1.

The crude extract was also analyzed by LC-UV-MS/MS. The LC-UV chromatogram (Figure 1) with diode array detection (DAD) shows the presence of other minor flavonoids such as compounds **3** (isoscutellarein), **5** (6-hydroxyluteolin), **6** (hispidulin), and **7** (luteolin) [28]–[31]. These compounds belong to the same flavone sub-class of flavonoids and produced UV-spectra with almost the same molar absorptivity at 335 nm. Therefore, the peaks at 9.8 (**1**) and 13.7 (**2**) min accounted majorly for the polyphenolic composition of the extract. The polyphenolic flavones could be identified by MS-MS ion scans. The collision of the precursor ions produced by ESI in the negative ion mode from the detected flavones yielded mass spectra for product ions; these spectra were used for their structural identification (Table 1). The most important product ions were produced by the retro-Diels-Alder type fragmentation at ring-C (dihydropyran ring), where the bond cleavage occurred at O(1)-C(2) and C(3)-C(4), resulting in anionic fragments containing ring A (*m/z* 151 and 167 respectively for di- and trihydroxylated ring A) and ring B (*m/z* 117 and 133 respectively for mono- and dihydroxylated ring B). Only compound **6** (hispidulin), which contains a methoxyl group at ring A, did not follow this fragmentation behavior. The first cleavage in this molecule is related to the loss of a radicalar methyl group; therefore, the path of fragmentation differed from the other flavones, and the retro-Diels-Alder fragmentation produced only minor fragments in its product ion spectrum.

### 3.2. Optimization of the Chromatographic Conditions

The compounds in the *A. chica* extract were analyzed using an HPLC gradient system, and good separation of the flavonoids, scutellarein and apigenin, was achieved within a short analysis time of 20 min. The flow rate of 1.0 mL/min allowed good separation, and the mobile phase was optimized using methanol in water containing 0.3% formic acid. The maximum absorption of the flavonoids was determined at 335 nm, and major absorption peaks were selected for analysis.

**Figure 2:**
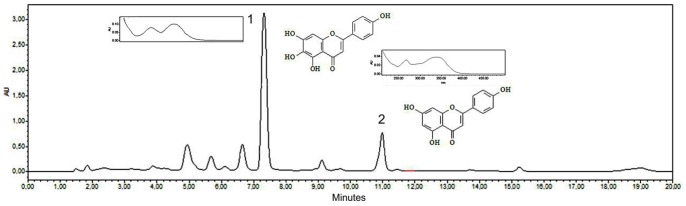
HPLC chromatogram of the *Arrabidaea chica* extract. Peak 1 (scutellarein) and peak 2 (apigenin).

Figure 2 shows the chromatogram of the *A. chica* extract. Peak 1 with a retention time of 7.33 min was identified as scutellarein (6-hydroxyapigenin), and peak 2 with a retention time of 10.98 min was considered to be apigenin.

### 3.3. Validation of Scutellarein and Apigenin

For the validation of the analytical method based on HPLC, the following factors were evaluated: linearity; precision; detection limits; and quantification and accuracy of the detection of the 2 substances found in the extract, scutellarein and apigenin. This validation was performed according to the ANVISA guidelines (resolution RE N° 899, 2003) [32].

#### 3.3.1. Linearity

The correlation coefficient of 0.9992 and 0.9938 showed a linear relationship between the corresponding peak areas for scutellarein and apigenin, respectively. The concentrations of scutellarein and apigenin were in the range of 31.25 to 500 µg/mL and 15.63 to 500 µg/mL, respectively. Table 2 describes the validation parameters of the calibration curve, including the linearity range, slope, intercept, and correlation coefficient obtained by linear regression analysis.

**Table 2:** Linearity parameters for the calibration curve of scutellarein and apigenin.

Compound	Linearity range(µg/mL)	Slope (a)	Intercept (b)	(r^2^)
Scutellarein	31.25–500	43,000	−6252.3	0.9992
Apigenin	15.63–500	67,357	74,8085	0.9938

r^2^, correlation coefficient.

#### 3.3.2. Precision

Repeatability and intermediate precision were evaluated by performing the analyses in triplicate for each concentration level of 31.25, 125, and 500 µg/mL for scutellarein and 15.63, 62.5, and 500 µg/mL for apigenin. The repeatability and intermediate precision tests showed RSD values of less than 3.27% and less than 4.15%, respectively, for scutellarein, and less than 2.21% and less than 2.02%, respectively, for apigenin (Table 3).

**Table 3:** Repeatability and intermediate precision data for the determination of scutellarein and apigenin by HPLC.

Compound	Concentration (µg/mL)	Repeatability (RSD%)^1^	Intermediate precision (RSD%)^1^
Scutellarein	31.25	3.27	3.02
	125	0.51	4.15
	500	2.42	2.78
Apigenin	15.63	2.014	1.375
	62.5	2.210	2.018
	500	0.324	0.484

1 RSD is the relative standard deviation for each sample (n = 3).

#### 3.3.3. Limit of quantification and detection

The LOQ, which was 27.88 µg/mL for scutellarein and 2.548 µg/mL for apigenin, was defined as the smallest quantity of the compound in a sample that could be quantified with acceptable precision and accuracy. The LOD, which was 8.37 µg/mL for scutellarein and 0.765 µg/mL for apigenin, was defined as the smallest quantity of the compound that could be detected in a sample, but not necessarily under the stated experimental conditions.

#### 3.3.4. Recovery

The accuracy of the analytical method based on HPLC was evaluated using the recovery test. The method produced a mean recovery of 104.54% with RSD below 1.13% for all analyzed concentrations of scutellarein and a mean recovery of 105.25% with RSD below 0.94% for apigenin, confirming the accuracy of the method (Table 4). In this analysis, a recovery between 70% and 120% was considered acceptable [33].

**Table 4:** Results of the recovery test for scutellarein and apigenin from the extract of *A. chica*.

Compound	Spiked concentration (µg/mL)	Recovery (%) (mean ± SD)^1^	Mean ± SD^1^	RSD^2^ (%)
Scutellarein	31.25	103.51±3.13	104.54±1.13	1.13
	125	104.29±1.46		
	500	105.82±2.68		
Apigenin	15.63	105.56±0.37	105.25±0.99	0.94
	62.5	104.15±3.32		
	250	106.05±4.28		

1 SD is the standard deviation for each sample (n = 3);

2 RSD is the relative standard deviation.

#### 3.3.5. Analysis of Leaves of *A. chica*

The retention time of standard apigenin was used to identify the corresponding peaks in the leaf extract of *A. chica,* and the apigenin content in the extract was evaluated using the regression equation y = 67357x+748085. The concentration of scutellarein was evaluated using the equation y = 43000x–6252.3 (Table 5).

**Table 5:** Quantification of scutellarein and apigenin in extract of leaves from *A. chica* by HPLC.

	Scutellarein (µg/mL) (mean ± RSD)	Apigenin (µg/mL) (mean ± RSD)
Leaves from *A. Chica*	818.60±3.26	112.04±0.52

RSD is the relative standard deviation for each sample (n = 3).

### 3.4. Antioxidant Capacity

Because of the multifunctional properties of phytochemicals, the antioxidant efficiency of a plant extract is measured on the basis of the results obtained by commonly accepted assays performed using different oxidative conditions, system compositions, and antioxidant mechanisms [34], [35]. Accordingly, in the present study, 2 spectrophotometric assays (DPPH method and β-carotene bleaching test) and a chemiluminescence method (TRAP method) were used to analyze the antioxidant capacity of the leaf extract of *A. chica* cultivated in Southern Brazil.

**Figure 3:**
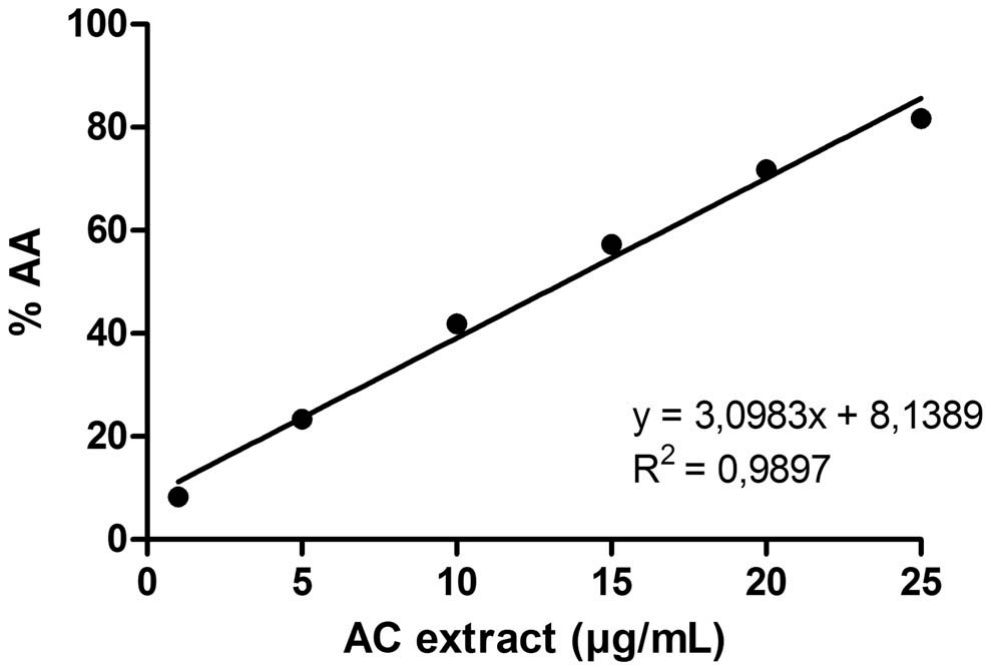
DPPH radical scavenging capacity of *Arrabidaea chica* (AC) extract. R^2^ = 0.9897.

The DPPH test has widely been used to test the ability of a compound to act as free radical scavenger or hydrogen donor and thus to evaluate its antioxidant activity [23], [36]–[37]. The leaf extract quenched DPPH free radicals in a dose-dependent manner. As the concentration of the *A. chica* extract increased, its DPPH quenching activity also increased (Figure 3).

The IC_50_ of the extract was 13.51 µg/mL, which is in accordance with the findings of Jorge et al. [38] who described significant antioxidant activity (IC_50_ = 15.98 µg/mL) of the extract of *A. chica* cultivated in Southern Brazil by using the DPPH method. However, other authors who used the *A. chica* plants cultivated in the Amazonian region of Brazil suggested a low antioxidant activity [35]. These results indicated that the antioxidant capacity of the extract collected from the same plant species can vary depending on the source and environment in which the plants were cultivated.

The extract of *A. chica* had an antioxidant capacity index of 2.23 and BHT value of 4.43; both values indicate a very strong antioxidant activity according to the classification of Scherer and Godoy [22].

The reaction between an antioxidant and DPPH depends on the structural conformation of the antioxidant. Some compounds react quickly with DPPH leading to the reduction of DPPH molecules equal to the number of hydroxyl groups [6], [39]. Previous studies have reported that, among flavonoids, quercetin, which contains 2 hydroxyl groups on the B-ring, had the highest antioxidant activity when the IC_50_ values of individual flavonol compounds were compared; this indicated that the number and patterns of hydroxyl substitutions on the B-ring were associated with the major antioxidant activity [37], [40]. The 2 flavonoids (apigenin and scutellarein) detected in the extract of *A. chica* had 1 hydroxyl group on B-ring and 2 and 3 hydroxyl groups on the A-ring, respectively, and these flavonoids showed a very strong antioxidant activity (AAI of the extract = 2.23), suggesting that the hydroxyl groups on the A-ring could be involved in increasing the antioxidant potential of the extract.

The β-carotene bleaching test showed that the addition of the *A.* *chica* extract and quercetin in different concentrations prevented the bleaching of β-carotene at different degrees by neutralizing the linoleate and other free radicals formed in the system [23], [41]. Membrane lipids are rich in unsaturated fatty acids that are the most susceptible to oxidation. Particularly, linoleic acid and arachidonic acid are the targets of lipid peroxidation. The inhibition of lipid peroxidation by antioxidants has been thought to be due to their free radical scavenging activities [3], [6]. Table 6 shows that the extract and quercetin had substantial antioxidant activities in this system at concentrations of 200 µg/mL and 500 µg/mL.

**Table 6:** Antioxidant activity of the extract of *A. chica* on β-carotene-linoleate system.

Sample	Concentration (µg/mL)	Antioxidant activity (%) (mean ± SD)^1^
Extract of *A. chica*	200 µg/mL	51.2±3.38
	500 µg/mL	94±4.61*
Quercetin	200 µg/mL	74.8±0.26
	500 µg/mL	99.1±4.27

1 SD is the standard deviation for each sample (n = 3).

* no significant difference when compared to quercetin (ANOVA test; *p*<0.05).

The TRAP method indicates the free-radical scavenging ability of antioxidants against peroxyl radicals via the hydrogen atom transfer pathway [5], [27], [42]. The results showed dose-dependent correlation between the increasing concentrations of *A. chica* extract and the TRAP obtained by trolox (hydro-soluble vitamin E) used as a standard antioxidant. Among the samples tested (crude extract and pure compounds, scutellarein and apigenin obtained from *A. chica* extract), the crude extract showed the highest antioxidant activity (Table 7). When the antioxidant activities of scutellarein and apigenin isolated from *A. chica* were compared, scutellarein showed higher antioxidant activity than apigenin, which is in agreement with the findings of Kandasamy and Rathinam [43]. They suggested that the higher antioxidant potential of scutellarein is attributed to the electronic parameters present on the ring of the molecular structure of scutellarein, enabling it to scavenge more reactive oxygen species.

**Table 7:** Antioxidant activity of the extract of *A. chica*, scutellarein, and apigenin by TRAP (µM Trolox).

Concentration (µg/mL)	Extract of *A. chica* (µM Trolox)(mean ± SD)^1^	Scutellarein (µM Trolox)(mean ± SD)	Apigenin (µM Trolox)(mean ± SD)
0.1 µg/mL	0.4738±0.04662	0.2804±0.001	0.1807±0.009
0.5 µg/mL	1.981±0.1603	1.846±0.073*	1.154±0.065
1.0 µg/mL	6.877±1.445	3.540±0.139	2.338±0.090

1 SD is the standard deviation for each sample (n = 3).

* no significant difference when compared to extract of *A. chica* (ANOVA test; *p*<0.05).

The TRAP method suggested that the significant antioxidant activity of the crude extract against the 2 metabolites obtained from *A. chica* might be because of the synergistic effect of the two major flavonoids present in the extract (scutellarein and apigenin), with other minor flavonoids (such as isoscutellarein, 6-hydroxyluteolin, hispidulin, and luteolin) that are known to have antioxidant activity [44]–[47]. The antioxidant activity of crude extract was significant and dose-dependent as revealed by TRAP, DPPH, and β-carotene/linoleic acid tests.

## Conclusions

The validation test suggested that our method had linearity, precision, and accuracy in the range studied, confirming that this method is appropriate for conducting quality control analysis of the extract and phytopharmaceutical and cosmetic preparations obtained from *A. chica.* Our HPLC method allowed the detection and quantification of 2 flavonoids (scutellarein and apigenin) from the extract obtained from the leaves of *A. chica.* To our knowledge, this is the first report of the extraction of scutellarein from this species.

The phenolic composition of *A. chica* leaves was determined by liquid chromatography coupled to diode array detection (LC–DAD) and liquid chromatography coupled to electrospray ionization-tandem mass spectrometry (LC–ESI-MS/MS), and isoscutellarein, 6-hydroxyluteolin, hispidulin, scutellarein, luteolin, and apigenin were identified.

The results from various antioxidant systems indicated that the extract of *A. chica* had significant antioxidant activity, even when the antioxidant capacity of the metabolites were compared separately using the TRAP method. This highest activity could be attributed to the presence of a mixture of compounds in the plant extract (isoscutellarein, 6-hydroxyluteolin, hispidulin, scutellarein, luteolin, and apigenin). We know that the yield of metabolites from plants is small; hence, it is economical to use the crude extract instead of the isolated metabolites Moreover, cosmetic products are developed using the crude extract for commercialization, because consumers require products developed from natural products with proven effectiveness as well as it is economical for industries to use the herbal extract. The significant antioxidant activity of the crude extract suggests that an antioxidant product with high amounts of plant components can be developed for use in the cosmetic industry.

Further investigations on individual compounds and their in vivo antioxidant activity must be performed in the future.
